# *Circ-MBOAT2* Regulates Angiogenesis via the *miR-495*/NOTCH1 Axis and Associates with Myocardial Perfusion in Patients with Coronary Chronic Total Occlusion

**DOI:** 10.3390/ijms25020793

**Published:** 2024-01-08

**Authors:** Wei Gao, Chenguang Li, Jie Yuan, Youming Zhang, Guobing Liu, Jianhui Zhang, Hongcheng Shi, Haibo Liu, Junbo Ge

**Affiliations:** 1 Department of Cardiology, Shanghai Institute of Cardiovascular Diseases, Zhongshan Hospital, Fudan University, Shanghai 200032, China; gao.wei1@zs-hospital.sh.cn (W.G.); li.chenguang@zs-hospital.sh.cn (C.L.);; 2 National Clinical Research Center for Interventional Medicine, Shanghai 200032, China; 3 Department of Cardiology, Qingpu Branch of Zhongshan Hospital Affiliated to Fudan University, Shanghai 201700, China; 4 Department of Nuclear Medicine, Zhongshan Hospital, Fudan University, Shanghai 200032, China

**Keywords:** *circ-MBOAT2*, *miR-495*, angiogenesis, chronic total occlusion, myocardial perfusion

## Abstract

Revascularization of coronary chronic total occlusion (CTO) still remains controversial. The factors that impact collateral circulation and myocardial perfusion are of interest. Circular RNA (circRNA) has been shown to regulate the process of angiogenesis. However, the effects of circ-membrane-bound O-acyltransferase domain containing 2 (*circ-MBOAT2*) on angiogenesis in patients with CTO were unclear. In this study, we evaluated circulating circRNAs and miRNAs in patients with CTO and stable coronary artery disease using high-throughput sequencing. Another cohort of patients were selected to verify the expressions of *circ-MBOAT2* and *miR-495*. The role and mechanism of *circ-MBOAT2* in the process of angiogenesis were explored through in vitro and vivo studies. Finally, we came back to a clinical perspective and investigated whether *circ-MBOAT2* and *miR-495* were associated with the improvement of myocardial perfusion evaluated by single-photon emission computed tomography (SPECT). We found that the expression of *circ-MBOAT2* was significantly up-regulated while *miR-495* was significantly down-regulated in patients with CTO. The expression of *circ-MBOAT2* was negatively correlated with *miR-495* in patients with CTO. In an in vitro study, we found that *circ-MBOAT2* promoted tube formation and cell migration via the *miR-495*/NOTCH1 axis in endothelial cells. In an in vivo study, we showed that the inhibition of *miR-495* caused the increase in collateral formation in mice after hindlimb ischemia. In a human study, we showed the expressions of *circ-MBOAT2* and *miR-495* were associated with myocardial perfusion improvement after revascularization of CTO. In conclusion, *circ-MBOAT2* regulates angiogenesis via the *miR-495*/NOTCH1 axis and associates with myocardial perfusion in patients with CTO. Our findings suggest that *circ-MBOAT2* and *miR-495* may be potential therapeutic targets and prognostic factors for patients with CTO.

## 1. Introduction

Cardiovascular disease remains the leading cause of death worldwide. Among patients with known coronary artery disease (CAD), the prevalence of chronic total occlusion (CTO) is between 30% and 50% [1,2]. CTO is defined as complete vessel occlusion of a native coronary artery, and the estimated occlusion duration is not less than 3 months. Despite the high prevalence, only a portion of these occlusions were treated with revascularization [3]. Clinical guidelines recommend that revascularization can be considered if ischemia reduction in the CTO territory and/or relief of angina symptoms can be expected [4]. Thus, other methods to improve myocardial perfusion and predictors to screen for potential candidates for revascularization are of interest to researchers. 

A specific characteristic of CTO is collateral circulation, which can be seen in almost all affected patients. The underlying mechanisms of angiogenesis are complex, including shear stress, molecular, and cellular responses to hypoxia. MicroRNAs (miRNAs) are small non-coding RNA molecules that play an important role in angiogenesis. The roles of miRNAs have been thoroughly investigated in patients with CAD [5,6]. Circular RNAs (circRNAs), a novel RNA with the ability in absorbing miRNAs, have been demonstrated to be widely involved in the process of angiogenesis in the tumor microenvironment [7]. However, the expression profiles and biological roles of miRNAs and circRNAs have not been investigated in patients with CTO. 

In this study, we identified differentially expressed miRNAs and circRNAs in patients with CTO and stable CAD. We found that *circ-MBOAT2* was up-regulated and *miR-495* was down-regulated in patients with CTO, especially in those with good collateral circulation. *miR-495* belongs to the 14q32 miRNA gene cluster, which was first discovered in 2004 [8] and found to be involved in post-ischemic blood flow recovery in mice [9]. Starbase online database shows that Notch1 contains the binding sequence of *miR-495* and *miR-495* contains the binding sites of *circ-MBOAT2*. Thus, we propose a hypothesis that *Circ-MBOAT2* regulates angiogenesis by *miR-495*/NOTCH1 axis in patients with CTO. 

## 2. Results

### 2.1. Expressions of Circ-MBOAT2 and miR-495 in Control, CAD and Patients with CTO

**Figure 1:**
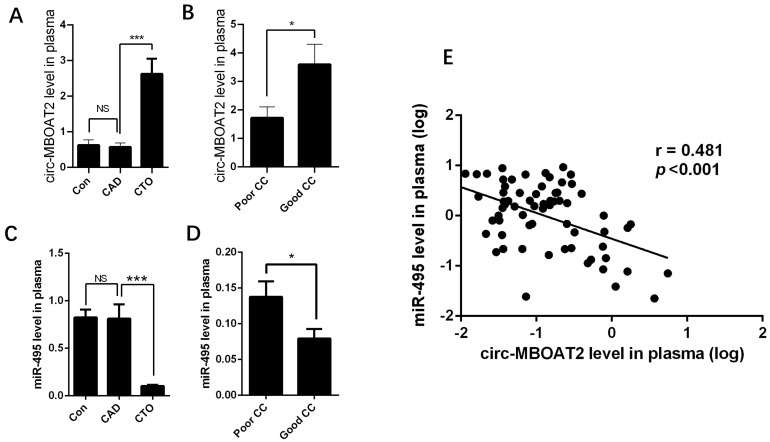
**Expressions of *circ-MBOAT2* and *miR-495* in control, CAD, and patients with CTO.** (**A**) Expression of *circ-MBOAT2* in control, CAD, and patients with CTO. (**B**) Patients in the CTO group were divided into two subgroups according to CC grade and *circ-MBOAT2* expression into good CC and poor CC groups. (**C**,**D**) *miR-495* expression in these patients grouped as above. (**E**) The association of *circ-MBOAT2* and *miR-495*. NS, non-significant; *, *p* < 0.05; ***, *p* < 0.001.

*Circ-MBOAT2* and *miR-495* expressions were determined in 146 patients, including 50 control patients without significant coronary stenosis confirmed by angiography, 50 patients with CAD with a 50–90% stenosis, and 46 patients with CTO. Results showed that *circ-MBOAT2* expression was significantly increased in patients with CTO compared with that in patients with CAD (Figure 1A). Then, we divided the patients with CTO into poor CC and good CC groups according to collateral grading. The results showed that *circ-MBOAT2* expression was significantly higher in the good CC group than that in the poor CC group (Figure 1B). The expression of *miR-495* was significantly decreased in patients with CTO (Figure 1C), especially in the good CC group (Figure 1D). In addition, linear correlation analysis demonstrated that *circ-MBOAT2* was negatively related to *miR-495* expression (Figure 1E). These results showed that *circ-MBOAT2* and *miR-495* might have a synergetic role in the process of angiogenesis. In the next steps, we conducted a series of experiments to confirm the possible roles and the underlying mechanisms of *circ-MBOAT2* and *miR-495* in angiogenesis. 

### 2.2. Circ-MBOAT2 Increases Tube Formation and Cell Migration in HUVEC

**Figure 2:**
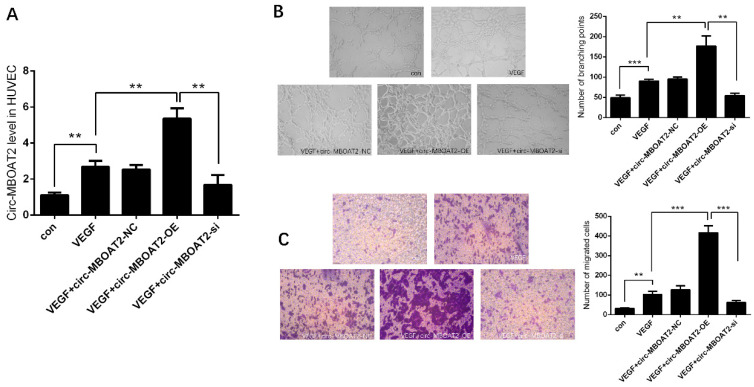
***Circ-MBOAT2* increases tube formation and cell migration in HUVEC.** (**A**) Expression of *circ-MBOAT2* in HUVEC treated with VEGF. (**B**,**C**) Impact of *circ-MBOAT2* knockdown and overexpression on tube formation and cell migration in HUVEC. **, *p* < 0.01; ***, *p* < 0.001.

We investigated the expression and the role of *circ-MBOAT2* in HUVEC. After treatment with VEGF (10 ng/mL), the *circ-MBOAT2* expression was significantly increased in HUVEC. The overexpression plasmids successfully increased the expression of *circ-MBOAT2*, and the si-*circ-MBOAT2* successfully silenced the expression of *circ-MBOAT2* (Figure 2A). The tube formation of HUVEC was increased by overexpression of *circ-MBOAT2* and was decreased by inhibition of *circ-MBOAT2*, respectively (Figure 2B). Similarly, the migration of HUVEC was also increased by overexpression of *circ-MBOAT2* and was decreased by inhibition of *circ-MBOAT2*, respectively (Figure 2C). These results indicate that *circ-MBOAT2* is involved in the process of angiogenesis. 

### 2.3. Circ-MBOAT2 Acted as a Sponge of miR-495

**Figure 3:**
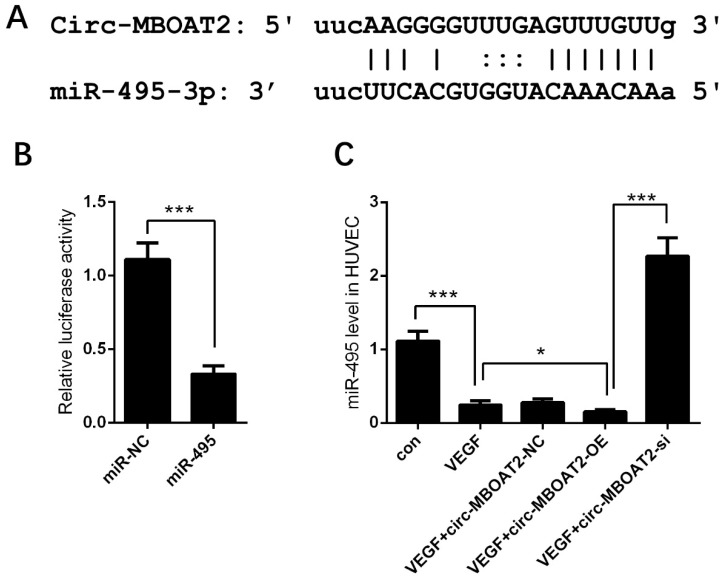
***Circ-MBOAT2* was associated with *miR-495* in HUVEC.** (**A**) The binding sites between *circ-MBOAT2* and *miR-495* were predicted by an online database. (**B**) The association between *circ-MBOAT2* and *miR-495* was illustrated by a dual-luciferase reporter assay. (**C**) Impact of *circ-MBOAT2* knockdown and overexpression on the expression of *miR-495* in HUVEC. *, *p* < 0.05; ***, *p* < 0.001.

The underlying mechanism by which *circ-MBOAT2* regulated angiogenesis awaits to be unveiled. Based on previous findings, we searched the online database and found the binding sequence between *miR-495* and *circ-MBOAT2* (Figure 3A). Dual-luciferase reporter assay showed that the relative luciferase activity was dramatically repressed in *circ-MBOAT2* and *miR-495* group; however, no significant change was observed in *circ-MBOAT2* and *miR-495* control group (Figure 3B). The effects of *circ-MBOAT2* on the expressions of *miR-495* were further investigated. We found that *miR-495* expression was significantly down-regulated by *circ-MBOAT2* and up-regulated by si-*circ-MBOAT2* (Figure 3C). These results show that *circ-MBOAT2* acts as a sponge of *miR-495*.

### 2.4. miR-495 Inhibits Angiogenesis through NOTCH1 Pathway in Endothelial Cells

**Figure 4:**
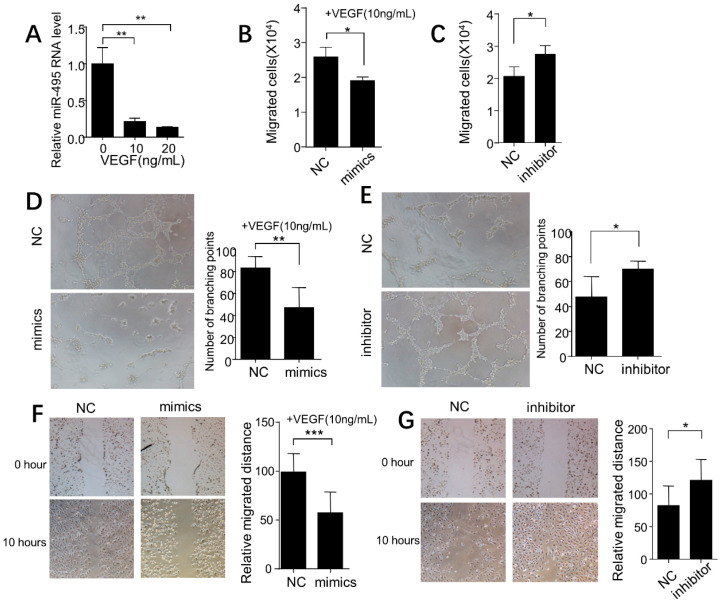
**Role of *miR-495* in angiogenesis of HUVECs.** (**A**) *miR-495* expression determined by RT-PCR in HUVECs treated with different concentrations of VEGF for 24 h. (**B**,**C**) Impact of *miR-495* mimics and inhibitor on the migration of HUVEC. (**D**,**E**) Impact of *miR-495* mimics and inhibitor on the tube formation of HUVEC. (**F**,**G**) Impact of *miR-495* mimics and inhibitor on the wound healing of HUVEC. *, *p* < 0.05; **, *p* < 0.01; ***, *p* < 0.001.

After treatment with VEGF (10 or 20 ng/mL), the *miR-495* expression was significantly inhibited (Figure 4A). Then, we investigated the role of *miR-495* in angiogenesis of HUVEC. As shown in Figure 4B,C, the transwell migration assay found that *miR-495* mimics inhibited cell migration and that *miR-495* inhibitor increased cell migration. Furthermore, *miR-495* mimics inhibited tube formation and *miR-495* inhibitor increased tube formation (Figure 4D,E). The cell wound scratch assay showed similar results (Figure 4F,G). 

**Figure 5:**
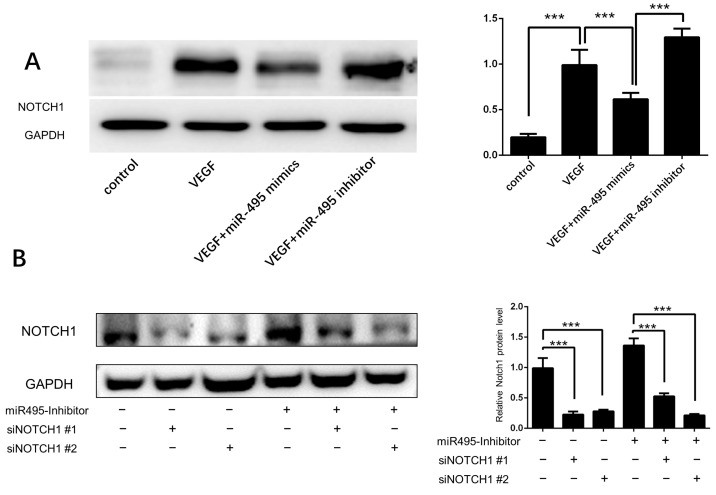
***miR-495* targets Notch1 in HUVECs.** (**A**) HUVECs were cultured with VEGF (10 ng/mL), *miR-495* mimics, or inhibitors for 48 h, and the protein level of Notch1 was determined by WB. (**B**) HUVECs were cultured with *miR-495* inhibitor or without. Then, siNotch1 #1 or #2 was added into medium, and the protein level of Notch1 was determined after 48 h. ***, *p* < 0.001.

**Figure 6:**
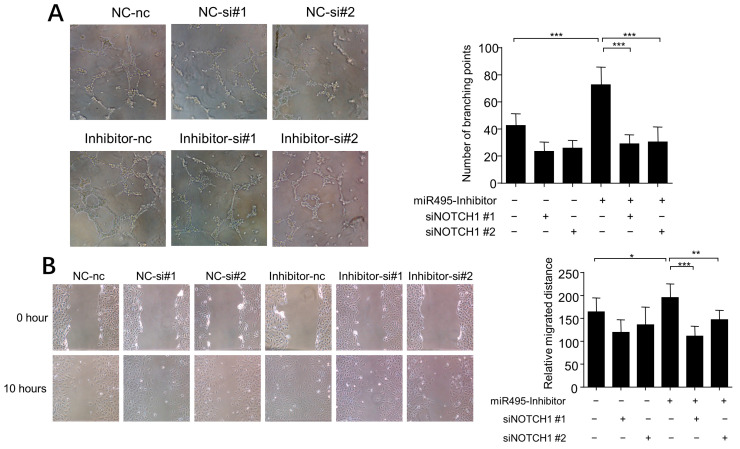
**Notch1 was involved in *miR-495* inhibitor-induced angiogenesis.** (**A**,**B**) HUVECs were cultured with *miR-495* inhibitor or without. siNotch1 NC #1 or #2 was added at a concentration of 50 nM. Formation of tube-like structures was recorded 8 h later (**A**). Wound healing assay was performed 10 h after scratch (**B**). *, *p* < 0.05; **, *p* < 0.01; ***, *p* < 0.001.

We used www.targetscan.org to predict targets of *miR-495* and found that Notch1 was one of the potential targets. We treated the HUVEC with VEGF and found that Notch1 expression was elevated. We also confirmed that *miR-495* mimics inhibited Notch1 expression and *miR-495* inhibitor increased Notch1 expression (Figure 5A). To assess the role of Notch1 in the association between *miR-495* and angiogenesis, we constructed two Notch1 siRNAs to interfere with the Notch1 expression. As shown in Figure 5B, both siNotch1 #1 and siNotch1 #2 successfully alleviated the increased expression of Notch1 induced by *miR-495* inhibitor. HUVEC were then treated with *miR-495* inhibitor and Notch1 siRNAs. Tube formation and wound scratch assay showed that *miR-495* inhibitor-induced angiogenesis of HUVEC was alleviated by Notch1 siRNAs (Figure 6A,B). These results demonstrate that *miR-495* inhibits angiogenesis through the NOTCH1 pathway in endothelial cells.

### 2.5. Inhibition of miR-495 Increases Collateral Formation in Mice after Hindlimb Ischemia 

**Figure 7:**
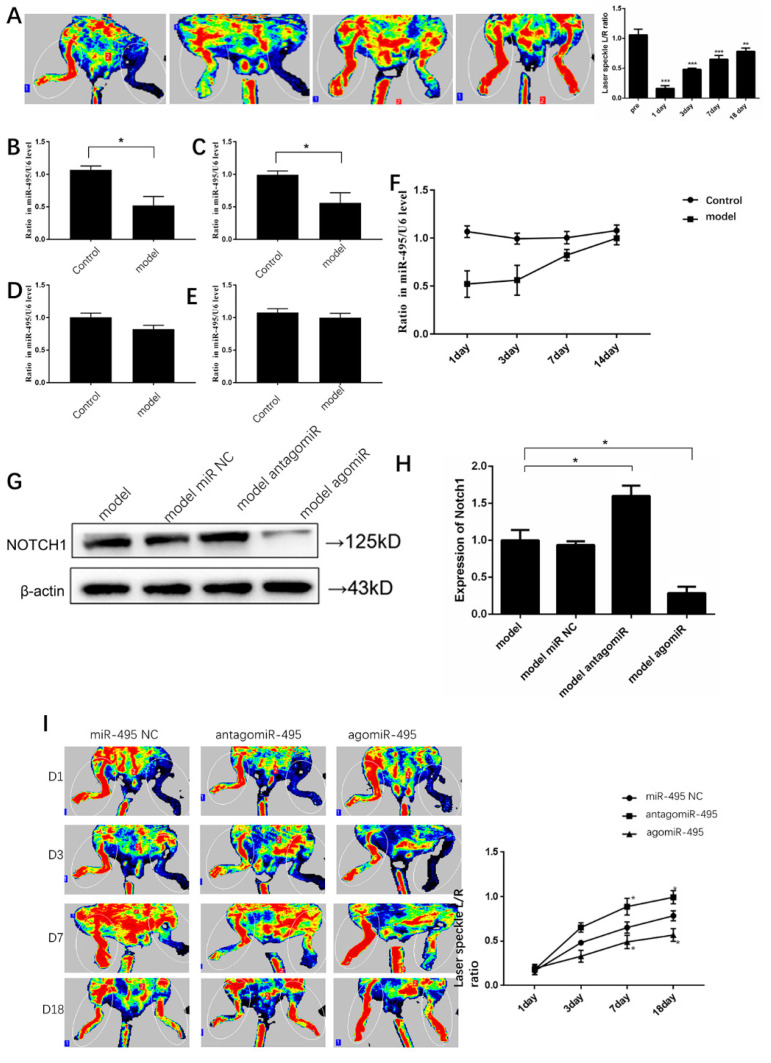
**Notch1 was the target of *miR-495* in mice hindlimb ischemia model.** (**A**) Representative pictures of blood flow in mice hindlimbs. The left femoral artery of mice was ligated and limb perfusion was assessed on day 1, 3, 7, and 18 via laser Doppler perfusion imaging. (**B**–**F**) After hindlimb ischemia, the expressions of *miR-495* in plasma were detected on day 1 (**B**), 3 (**C**), 7 (**D**), and 14 (**E**). A gradual recovery of *miR-495* expression was also shown (**F**). (**G**,**H**) After ligation, *miR-495* NC, antago-*miR-495*, and ago-*miR-495* were injected into the ischemic muscles every other day. Then, we detected the expressions of Notch1 protein on day 3. (**I**) Representative pictures of blood flow in mice hindlimbs. The left femoral arteries of mice were ligated and *miR-495* NC, antago-*miR-495*, and ago-*miR-495* were injected into the ischemic muscles. Limb perfusion was assessed on day 1, 3, 7, and 18 via laser Doppler perfusion imaging. *, *p* < 0.05; **, *p* < 0.01; ***, *p* < 0.001. *n* = five mice in each group.

To explore the roles of *miR-495* in collateral formation in mice, we performed a hindlimb ischemia study. After the left femoral artery ligation, the blood flow in ischemic hindlimbs, measured using laser doppler perfusion, began to recover obviously on day 3 (Figure 7A). Meanwhile, plasma *miR-495* expressions after hindlimb ischemia were determined at different time points. We found that *miR-495* was significantly down-expressed one day after hindlimb ischemia (Figure 7B). From day 3 to day 14, its expression gradually recovered to a nearly normal level (Figure 7C–F). Notch1 was a target of *miR-495* and it has been shown to be involved in angiogenesis in previous HUVEC experiments. After ligation, *miR-495* antago and *miR-495* ago were injected into ischemic muscles. Then, we detected expression of Notch1 and found that antago*miR-495* increased Notch1 expression and ago*miR-495* decreased Notch1 expression (Figure 7G,H). To investigate whether *miR-495* plays a role in ischemia-mediated collateral formation in the hindlimb, we performed a blood flow study. The results showed that collateral formation after ischemia was enhanced by antago*miR-495* and inhibited by ago*miR-495* (Figure 7I). These findings suggest that *miR-495* was involved in collateral formation in mice after hindlimb ischemia and that Notch1 is the target of *miR-495*.

### 2.6. Circ-MBOAT2 Regulated Notch1 Expression and Angiogenesis by Absorbing miR-495

**Figure 8:**
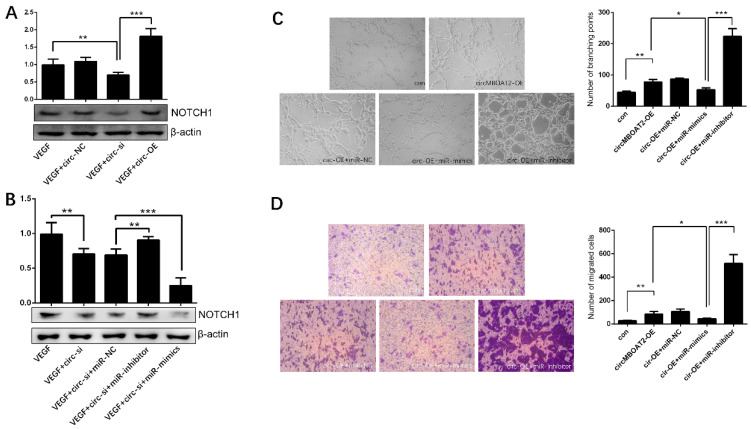
***Circ-MBOAT2* regulated Notch1 expression and angiogenesis by absorbing *miR-495*.** (**A**) Expression of Notch1 in HUVEC treated with *circ-MBOAT2* knockdown and overexpression. (**B**) Expression of Notch1 in HUVEC treated with *circ-MBOAT2* knockdown plus *miR-495* mimics or inhibitor. (**C**) Tube formation of HUVEC treated with *circ-MBOAT2* expression plus *miR-495* mimics or inhibitor. (**D**) Cell migration of HUVEC treated with *circ-MBOAT2* expression plus *miR-495* mimics or inhibitor. *, *p* < 0.05; **, *p* < 0.01; ***, *p* < 0.001.

We have demonstrated that *circ-MBOAT2* is a sponge of *miR-495* which targets NOTCH1. Next, we further revealed whether *circ-MBOAT2* regulates NOTCH1 expression by sponging *miR-495*. First, we showed that the expression of NOTCH1 was downregulated by inhibition of *circ-MBOAT2* and up-regulated by overexpression of *circ-MBOAT2* in HUVEC (Figure 8A). Then, we found *miR-495* inhibitors attenuated the down-regulation of NOTCH1 by inhibition of *circ-MBOAT2* (Figure 8B). Correspondently, the overexpression of *circ-MBOAT2* increased tube formation and cell migration (Figure 8C,D), and *miR-495* mimics weakened these effects (Figure 8C,D). These results indicate that *circ-MBOAT2* regulates angiogenesis via the *miR-495*/NOTCH1 axis. 

### 2.7. The Expressions of circ-MBOAT2 and miR-495 Are Associated with Myocardial Perfusion Improvement after Revascularization of CTO 

**Figure 9:**
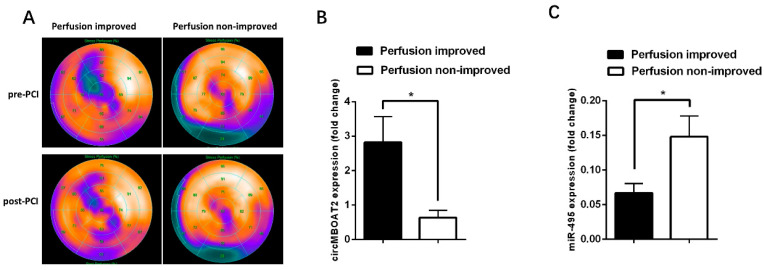
**Expressions of *circ-MBOAT2* and *miR-495* in patients with CTO with and without myocardial perfusion improvement after revascularization.** (**A**) Representative images of patients with improved (left panel) or not improved (right panel) perfusion after revascularization. (**B**,**C**) These 20 patients were divided into two groups according to whether myocardial perfusion was improved (*n* = 10 for improved group; *n* = 10 for non-improved group). Expressions of *circ-MBOAT2* and *miR-495* were determined, respectively. *, *p* < 0.05.

SPECT has been widely used to assess myocardial perfusion in patients with CAD, including patients with CTO. In this preliminary study, a total of 20 patients underwent SPECT both before revascularization of CTO and within 3 days after revascularization. For these patients, we investigated whether myocardial perfusion was associated with the expressions of *circ-MBOAT2* and *miR-495*. The patients were divided into two groups according to whether myocardial perfusion was improved after revascularization of CTO (Figure 9A). We found that the expression of *circ-MBOAT2* was significantly higher in patients with improved myocardial perfusion than in those patients without (Figure 9B). And for *miR-495*, the expression was significantly lower in patients with improved myocardial perfusion than in those patients without (Figure 9C). These preliminary findings indicate that expressions of *circ-MBOAT2* and *miR-495* are associated with myocardial perfusion improvement after revascularization and that they are potential biomarkers for guiding the revascularization strategy for patients with CTO. 

## 3. Discussion

In this study, we found that *circ-MBOAT2* and *miR-495* were differentially expressed in patients with CAD and CTO. *Circ-MBOAT2* regulated angiogenesis via the *miR-495*/NOTCH1 axis in in vitro and in vivo studies. Then, in human studies, we showed that the expressions of *circ-MBOAT2* and *miR-495* were associated with myocardial perfusion improvement after revascularization of CTO. 

Chemokines pathways have long been known to play an important role in the pathophysiological development of cardiovascular diseases [10]. As a sub-type of cardiovascular disease, CTO has received a lot of attention from scientific researchers. The specific characteristic of CTO is collateral circulation, which has attracted much attention in CTO research. Clinical practitioners focused on whether collateral circulation is sufficient in providing blood supply and whether it could predict clinical prognosis. Some other researchers were interested in the underlying mechanisms and impact factors of collateral circulation. In a previous study, we explored the relations between collateral circulation and some chemokines, such as thrombospondin-1, endostatin, and angiopoietin-2 [11]. We found that circulatory endostatin might be a useful biomarker for coronary collateral development and a potential target for therapeutic angiogenesis. Other studies also found some collateral-associated factors, such as gamma glutamyl transferase [12], neutrophil/lymphocyte ratio [13,14], C-reactive protein [15], mimecan [16], angiogenin, and osteopontin [17]. However, none of these previous studies further investigated the mechanisms by which these factors influence collateral circulation or their clinical significance. 

Mi-RNAs have been demonstrated to play various roles in cardiovascular diseases [18], including the coronary collateral circulation in patients with CTO [19]. A previous study identified some differentially expressed miRNAs in patients with CTO with well- or poorly developed collateral circulation [20]. There were some differences between their results and ours. This might be attributed to the inclusion of different participants; the previous study focused on patients with CTO with well- or poorly developed collateral circulation, whereas our study enrolled patients with CTO and CAD. In the past decade, the role of miRNAs in the regulation of both angiogenesis and arteriogenesis has been demonstrated. We observed a down-regulated expression of *miR-495* in patients with CTO and then we showed that *miR-495* inhibited angiogenesis through the NOTCH1 pathway. 

CircRNAs are a novel and evolutionarily conserved class of non-coding RNAs generated via an alternative RNA splicing approach termed “back-splicing” [21]. The characteristic feature of circRNAs is a close loop, and the main mechanism of action identified is through “sponging” target miRNAs [22]. Previous studies demonstrated that circRNAs were associated with angiogenesis [23,24] and that *circ-MBOAT2* acted as an oncogene in tumor growth [25,26]. The association between circRNA and miRNA was also confirmed in other ischemic diseases, such as ischemic stroke [27]. In this study, we determined the expression of *circ-MBOAT2* in patients with CTO and found that *circ-MBOAT2* was up-regulated in patients with CTO, especially in those with good collateral circulations. These findings encouraged us to further explore the underlying mechanism by which *circ-MBOAT2* regulates angiogenesis. Subsequent experiments showed that *circ-MBOAT2* regulated angiogenesis via the *miR-495*/NOTCH1 axis. 

SPECT has been widely accepted as the reference standard method for ischemia and viability testing in patients with CTO. A previous study showed that SPECT was helpful in terms of distinguishing patients who benefited the most from revascularization therapy [28]. To the best of our knowledge, no biomarkers have been developed for predicting myocardial perfusion improvement after revascularization in patients with CTO. In this study, we found that the myocardial perfusion improvement was associated with the expressions of *circ-MBOAT2* and *miR-495*, indicating that they are potential biomarkers for guiding the revascularization strategy for patients with CTO. Further clinical studies with large sample sizes are required to investigate the clinical significance of *circ-MBOAT2* and *miR-495* in patients with CTO. In a previous fundamental study, the revascularization of patients with CAD was associated with a significant reduction of cardiac death in those with >10% myocardial ischemia [29]. A threshold of 10% (or 5% with some other conditions) myocardial ischemia was then adopted in the ISCHEMIA study to determine whether patients should receive revascularization [30]. We believed that the combination of *circ-MBOAT2* and *miR-495* expressions may add prognostic value for patients with CTO. 

In conclusion, *circ-MBOAT2* regulates collateral formation and predicts myocardial perfusion via the *miR-495*/NOTCH1 axis in patients with CTO. With this study, we have further developed the knowledge of *circ-MBOAT2* and *miR-495* in both basic and clinical research of CTO diseases. Large translational studies are warranted in order to investigate whether *circ-MBOAT2* and *miR-495* have a prognostic role for patients with CTO. 

## 4. Materials and Methods

### 4.1. Patient Selection

As we previously reported [31], a total of 10 patients (5 with CAD and 5 with CTO) were selected for deep RNA and miRNA sequencing. Another 146 patients were selected to verify expressions of *miR-495* and *circ-MBOAT2*, including 50 control patients without significant coronary stenosis confirmed by angiography, 50 patients with CAD with a 50–90% stenosis, and 46 patients with CTO. CTO was defined as completely occluded coronary arteries with thrombolysis in myocardial infarction 0 flow with an estimated duration of at least 3 months [32]. The exclusion criteria were as follows: symptomatic peripheral arterial disease, recent acute myocardial infarction during last 3 months, decompensated heart failure, any concomitant inflammation or infectious diseases, neoplastic diseases, and severe liver and kidney dysfunctions. The cardiac history and risk factors of all patients were documented. A venous blood sample was collected from all patients upon admission (within 24 h). The blood sample was centrifuged at 1500× *g* for 10 min to precipitate blood cells, and plasma was then frozen at −80 °C until use.

This study was approved by the Medical Ethics Committee of Zhongshan Hospital, Fudan University. Informed consent was obtained from all patients. All procedures performed in the study were in accordance with the ethical standards of the institutional and/or national research committee and with the Helsinki declaration and its later amendments.

### 4.2. Collateral Grading 

Angiograms were reviewed by an experienced cardiologist who was blinded to the miRNA and circRNA analysis findings. Collateral vessels were classified by the CC grade [33]: CC0, no continuous connection between the donor and recipient arteries; CC1, continuous, thread-like connection (diameter: ≤0.3 mm); and CC2: continuous, small, side-branch-like size of the collateral throughout its course (diameter: ≥0.4 mm). The included patients with CTO were divided into poor CC (CC0–1) and good CC (CC2) groups. 

### 4.3. RNA Sequencing

Small RNAs (including miRNAs and circRNAs) were isolated from the total RNA of the patients’ plasma samples. High-throughput sequencing was performed by WuXi Next CODE (Shanghai, China) as previously described [31].

### 4.4. Cell Culture and Transfection

HUVECs were purchased from ATCC and cultured as described previously [34]. Briefly, the cells were cultured in a special medium (ECM; ScienCell, San Diego, CA, USA). For the experiments, VEGF was purchased from Peprotech (Cranbury, NJ, USA) and added to the medium at a concentration of 10 or 20 ng/mL for 24 h. MiRNA-495 mimics (50 nM, 5′-AAACAAACAUGGUGCACUUCUU-3′) and inhibitors (100 nM, 5′-AAGAAGUGCACCAUGUUUGUUU-3′) and NC (50 nM, 5′-CAGUACUUUUGUGUAGUACAA-3′) were transfected into the HUVECs using Lipo3000 according to the manufacturer’s instructions. Notch1 expression was knocked down using Notch siRNA (siNotch1 #1, 5′-CCAACUGCCAGACCAACAUTT-3′; siNotch1 #2, 5′-GGAUCCACUGUGAGAACAATT-3′). All oligos, including the overexpression plasmids and siRNA of *circ-MBOAT2*, were purchased from GenePharma (Shanghai, China). 

### 4.5. Measurement of HUVEC Migration and Tube Formation

HUVEC transwell migration assay was conducted using chambers with filters (pore size: 8 μm) coated with Matrigel (BD Biosciences, Franklin Lakes, NJ, USA). The cells (1 × 10^5^ cells per well) were seeded into the upper chamber, and invasive cells were harvested after incubation for 48 h. Wound healing assay was also performed to evaluate the migration ability. The cells were cultured in 6-well plates to reach 90% confluence. The cell monolayers were scraped using a 100-μL pipette tip, washed twice with PBS, and cultured for 24 h before being photographed. For the tube formation study, the HUVECs (2 × 10^4^ cells per well) were seeded onto Matrigel. The cells were microscopically recorded for the formation of tube-like structures 8 h later. For the NC or mimic group, 10 ng/mL VEGF was added.

### 4.6. Reverse Transcription-PCR

Total RNA was extracted from the cells, plasma, or tissues using TRIzol reagent (Invitrogen; Thermo Fisher Scientific, Waltham, MA, USA) according to the manufacturer’s protocol. The *miR-495* and *circ-MBOAT2* from each sample was quantified using SYBR Premix Ex Taq qRT-PCR assay (TaKaRa, Somerset, NJ, USA). Real-time PCR was performed using an ABI 7500 real-time PCR system. The relative expression levels of the miRNAs were normalized to that of U6 using the 2--ΔΔCq cycle threshold method. 

### 4.7. Western Blot Analysis

The cultured cells or tissues were harvested and lysed in RIPA buffer supplemented with complete protease inhibitor cocktail tablets. Cell debris was removed via centrifugation at 12,000 rpm for 30 min. The lysates were separated using SDS-PAGE, transferred to PVDF membranes (Bio-Rad, Hercules, CA, USA), and incubated with the relevant antibodies as indicated. Antibodies against Notch1 were purchased from Abcam (ab52627) (Cambridge, MA, USA).

### 4.8. Murine Hindlimb Ischemia Model

This study was approved by the institutional review boards of Zhongshan Hospital, Fudan University, and Shanghai Institutes for Biological Sciences. It was conducted in conformity with the Public Health Service Policy on Humane Care and Use of Laboratory Animals. Male C57BL/6 mice (age: 8–10 weeks) were used for the hindlimb ischemia model. Briefly, the mice were anesthetized with pentobarbital sodium (0.5%, 50 mg/kg) via intra peritoneal injection, and the surgical procedures were performed under sterile conditions. A vertical longitudinal incision was created in the left hindlimb; later, the femoral artery and its branches were dissected and ligated. For the right hindlimb (the non-ischemic control), a vertical longitudinal incision was also created; however, the femoral artery was not ligated.

### 4.9. In Vivo Transfection 

*miR-495* NC, ago, and antago oligonucleotides were purchased from Ribobio (Guangzhou, China). The male C57BL/6 mice received *miR-495* NC (5 nM), ago*miR-495* (5 nM), and antago*miR-495* (10 nM) via multi-point injections to the left adductors or gastrocnemius immediately after the operation.

### 4.10. Hindlimb Blood Flow Measurement

Hindlimb blood flow was measured using an imaging device with laser Doppler perfusion imaging on days 1, 3, 7, and 18 after the operation. The mice were anesthetized and placed on a 37 °C heating plate for 5 min. Blood flow was measured from the scanning images, and the perfusion ratio of the ischemic limbs was quantified by averaging the relative units of flux from the knee to the toe compared with that of the non-ischemic limbs (PIM Soft 1.4; Perimed, Sweden) [35].

### 4.11. SPECT among the Patients with CTO

SPECT was performed using a single-day rest/stress imaging protocol, as previously described [36]. Briefly, for rest imaging, pre-scanning was performed after the administration of an initial dose of approximately 1 mCi MIBI. Full scanning was started immediately after the injection of the remaining dose of approximately 15 mCi MIBI, and dynamic images were acquired in list mode for over 6 min. Following the rest of the dynamic scanning, rest perfusion scanning was performed. For stress imaging, pharmacological stress was induced via an intravenous infusion of adenosine triphosphate (ATP) disodium at a rate of 140 µg·kg^−1^·min^−1^ for 5 min, and 25 mCi MIBI was injected 3 min after the start of ATP injection, followed by dynamic image acquisition for over 6 min. Stress perfusion scanning was then performed. The myocardial ischemic percentage was calculated quantitatively by an independent doctor who was experienced in this specialty. Improved myocardial perfusion was defined as a reduction of 1% or more in myocardial ischemia after revascularization compared with the baseline. Image acquisition was performed using a D-SPECT cardiac scanner (Spectrum Dynamics, Caesarea, Israel).

### 4.12. Statistical Analyses

Data were presented as means ± standard deviations. For comparisons between two groups, a two–tailed Student’s *t*-test was used for parametric data and the Mann–Whitney U test for non-parametric data. For comparisons of n groups, one-way ANOVA was used for normally distributed variables. The linear relationship between *circ-MBOAT2* and *miR-495* was compared with Spearman’s correlation test. For all statistical analyses, significance was accepted at a 95% confidence level (*p* < 0.05). Calculations were performed using GraphPad Prism 6 or SPSS 21.0.

## 5. Limitations

There are some limitations of our study. First, the process of angiogenesis is complex and regulated by various factors. We used www.targetscan.org to predict targets of *miR-495* and found that Notch1 was one of the targets. Some other potential targets might be involved in the process of *miR-495*-regulated angiogenesis and will need further investigation in the future. Second, this study mainly focused on the basic research of *miR-495* and *circ-MBOAT2* in angiogenesis and only included a small sample of patients. The expression profiles and prognostic value of *miR-495* and *circ-MBOAT2* in patients with CTO should be explored in clinical studies with larger sample sizes. 

## Data Availability

All data are available from the corresponding author on reasonable request.

## References

[1] Christofferson R.D., Lehmann K.G., Martin G.V., Every N., Caldwell J.H., Kapadia S.R. (2005). Effect of chronic total coronary occlusion on treatment strategy. Am. J. Cardiol..

[2] Srinivas V.S., Brooks M.M., Detre K.M., King S.B., Jacobs A.K., Johnston J., Williams D.O. (2002). Contemporary percutaneous coronary intervention versus balloon angioplasty for multivessel coronary artery disease: A comparison of the National Heart, Lung and Blood Institute Dynamic Registry and the Bypass Angioplasty Revascularization Investigation (BARI) study. Circulation.

[3] Fefer P., Knudtson M.L., Cheema A.N., Galbraith P.D., Osherov A.B., Yalonetsky S., Gannot S., Samuel M., Weisbrod M., Bierstone D. (2012). Current perspectives on coronary chronic total occlusions: The Canadian Multicenter Chronic Total Occlu-sions Registry. J. Am. Coll. Cardiol..

[4] Windecker S., Kolh P., Alfonso F., Collet J.P., Cremer J., Falk V., Filippatos G., Hamm C., Head S.J., Authors/Task Force members (2014). 2014 ESC/EACTS Guidelines on myocardial revascularization: The Task Force on Myocardial Revascularization of the European Society of Cardiology (ESC) and the European Association for Cardio-Thoracic Surgery (EACTS)Developed with the special contribution of the European Association of Percutaneous Cardiovascular Interventions (EAPCI). Eur. Heart J..

[5] Chistiakov D.A., Orekhov A.N., Bobryshev Y.V. (2016). Cardiac-specific miRNA in cardiogenesis, heart function, and cardiac pathology (with focus on myocardial infarction). J. Mol. Cell. Cardiol..

[6] Economou E.K., Oikonomou E., Siasos G., Papageorgiou N., Tsalamandris S., Mourouzis K., Papaioanou S., Tousoulis D. (2015). The role of microRNAs in coronary artery disease: From pathophysiology to diagnosis and treatment. Atherosclerosis.

[7] Zhang Q., Wang W., Zhou Q., Chen C., Yuan W., Liu J., Li X., Sun Z. (2020). Roles of circRNAs in the tumour microenvironment. Mol. Cancer.

[8] Seitz H., Royo H., Bortolin M.-L., Lin S.-P., Ferguson-Smith A.C., Cavaillé J. (2004). A large imprinted microRNA gene cluster at the mouse Dlk1-Gtl2 domain. Genome Res..

[9] Welten S.M., Bastiaansen A.J., de Jong R.C., de Vries M.R., Peters E.A., Boonstra M.C., Sheikh S.P., La Monica N., Kandimalla E.R., Quax P.H. (2014). Inhibition of 14q32 MicroRNAs miR-329, miR-487b, miR-494, and *miR-495* increases neovascularization and blood flow recovery after ischemia. Circ Res..

[10] Márquez A.B., van der Vorst E.P.C., Maas S.L. (2021). Key Chemokine Pathways in Atherosclerosis and Their Therapeutic Potential. J. Clin. Med..

[11] Qin Q., Qian J., Ma J., Ge L., Ge J. (2016). Relationship between thrombospondin-1, endostatin, angiopoietin-2, and coronary collateral development in patients with chronic total occlusion. Medicine.

[12] Sahin M., Demir S., Kalkan M.E., Ozkan B., Alici G., Cakalagaoglu K.C., Yazicioglu M.V., Sarikaya S., Biteker M., Turkmen M.M. (2013). The relationship between gamma-glutamyltransferase and coronary collateral circulation in patients with chronic total occlusion. Anadolu Kardiyol. Derg./Anatol. J. Cardiol..

[13] Nacar A.B., Erayman A., Kurt M., Buyukkaya E., Karakaş M.F., Akcay A.B., Buyukkaya S., Sen N. (2014). The relationship between coronary collateral circulation and neutrophil/lymphocyte ratio in patients with coronary chronic total occlusion. Med. Princ. Pract..

[14] Kalkan M., Şahin M., Kalkan A., Güler A., Taş M., Bulut M., Demir S., Acar R., Arslantaş U., Öztürkeri B. (2014). The relationship between the neutrophil–lymphocyte ratio and the coronary collateral circulation in patients with chronic total occlusion. Perfusion.

[15] Söğüt E., Kadı H., Karayakalı M., Mertoğlu C. (2015). The association of plasma vitamin A and E levels with coronary collateral circulation. Atherosclerosis.

[16] Shen Y., Ding F.H., Zhang R.Y., Zhang Q., Lu L., Shen W.F. (2016). Association of serum mimecan with angiographic coronary collateralization in patients with stable coronary artery disease and chronic total occlusion. Atherosclerosis.

[17] Gurses K.M., Yalcin M.U., Kocyigit D., Besler M.S., Canpinar H., Evranos B., Yorgun H., Şahiner M.L., Kaya E.B., Özer N. (2019). The association between serum angiogenin and osteopontin levels and coronary collateral circulation in patients with chronic total occlusion. Anatol. J. Cardiol..

[18] Kabłak-Ziembicka A., Badacz R., Okarski M., Wawak M., Przewłocki T., Podolec J. (2023). Cardiac microRNAs: Diagnostic and therapeutic potential. Arch. Med. Sci..

[19] Papageorgiou N., Zacharia E., Tousoulis D. (2016). Association between microRNAs and coronary collateral circulation: Is there a new role for the small non-coding RNAs?. Ann. Transl. Med..

[20] Hakimzadeh N., Nossent A.Y., van der Laan A.M., Schirmer S.H., de Ronde M.W.J., Pinto-Sietsma S.-J., van Royen N., Quax P.H.A., Hoefer I.E., Piek J.J. (2015). Circulating MicroRNAs Characterizing Patients with Insufficient Coronary Collateral Artery Function. PLoS ONE.

[21] Hou L.-D., Zhang J. (2017). Circular RNAs: An emerging type of RNA in cancer. Int. J. Immunopathol. Pharmacol..

[22] Najafi S. (2022). Circular RNAs as emerging players in cervical cancer tumorigenesis; A review to roles and biomarker potentials. Int. J. Biol. Macromol..

[23] Ghaedrahmati F., Nasrolahi A., Najafi S., Mighani M., Anbiyaee O., Haybar H., Assareh A.R., Kempisty B., Dzięgiel P., Azizidoost S. (2023). Circular RNAs-mediated angiogenesis in human cancers. Clin. Transl. Oncol..

[24] Ma X., Chen X., Mo C., Li L., Nong S., Gui C. (2022). The role of circRNAs in the regulation of myocardial angiogenesis in coronary heart disease. Microvasc. Res..

[25] Zhou X., Liu K., Cui J., Xiong J., Wu H., Peng T., Guo Y. (2021). *Circ-MBOAT2* knockdown represses tumor progression and glutamine catabolism by miR-433-3p/GOT1 axis in pancreatic cancer. J. Exp. Clin. Cancer Res..

[26] Shi J., Liu C., Chen C., Guo K., Tang Z., Luo Y., Chen L., Su Y., Xu K. (2020). Circular RNA circMBOAT2 promotes prostate cancer progression via a miR-1271-5p/mTOR axis. Aging.

[27] Zhuang Y., Fan W.P., Yan H.S. (2023). Overexpression of Circ_0005585 Alleviates Cerebral Ischemia Reperfusion Injury via Targeting MiR-16-5p. Bull. Exp. Biol. Med..

[28] Sun D., Wang J., Tian Y., Narsinh K., Wang H., Li C., Ma X., Wang Y., Wang D., Li C. (2012). Multimodality imaging evaluation of functional and clinical benefits of percutaneous coronary intervention in patients with chronic total occlusion lesion. Theranostics.

[29] Hachamovitch R., Hayes S.W., Friedman J.D., Cohen I., Berman D.S. (2003). Comparison of the short-term survival benefit associated with revascularization compared with medical therapy in patients with no prior coronary artery disease undergoing stress myocardial perfusion single photon emission computed tomography. Circulation.

[30] Maron D.J., Hochman J.S., Reynolds H.R., Bangalore S., O’Brien S.M., Boden W.E., Chaitman B.R., Senior R., López-Sendón J., Alexander K.P. (2020). Initial Invasive or Conservative Strategy for Stable Coronary Disease. N. Engl. J. Med..

[31] Gao W., Zhang J., Wu R., Yuan J., Ge J. (2022). Integrated Analysis of Angiogenesis Related lncRNA-miRNA-mRNA in Patients with Coronary Chronic Total Occlusion Disease. Front Genet..

[32] Brilakis E.S., Mashayekhi K., Tsuchikane E., Rafeh N.A., Alaswad K., Araya M., Avran A., Azzalini L., Babunashvili A.M., Bayani B. (2019). Guiding Principles for Chronic Total Occlusion Percutaneous Coronary Intervention. Circulation.

[33] Meisel S.R., Frimerman A., Blondheim D.S., Shotan A., Asif A., Shani J., Shochat M. (2013). Relation of the systemic blood pressure to the collateral pressure distal to an infarct-related coronary artery occlusion during acute myocardial infarction. Am. J. Cardiol..

[34] Gao W., Liu H., Yuan J., Wu C., Huang D., Ma Y., Zhu J., Ma L., Guo J., Shi H. (2016). Exosomes derived from mature dendritic cells increase endothelial inflammation and atherosclerosis via membrane TNF-alpha mediated NF-kappaB pathway. J. Cell. Mol. Med..

[35] Bai Y., Liu R., Li Z., Zhang Y., Wang X., Wu J., Li Z., Qian S., Li B., Zhang Z. (2019). VEGFR endocytosis regulates the angiogenesis in a mouse model of hindlimb ischemia. J. Thorac. Dis..

[36] Li C., Xu R., Yao K., Zhang J., Chen S., Pang L., Lu H., Dai Y., Qian J., Shi H. (2020). Functional significance of intermediate coronary stenosis in patients with single-vessel coronary artery disease: A comparison of dynamic SPECT coronary flow reserve with intracoronary pressure-derived fractional flow reserve (FFR). J. Nucl. Cardiol..

